# Dehydration-associated chronic kidney disease: a novel case of kidney failure in China

**DOI:** 10.1186/s12882-020-01804-x

**Published:** 2020-05-04

**Authors:** Xinglin Yang, Haiting Wu, Hang Li

**Affiliations:** 1 Department of Internal Medicine, Peking Union Medical College Hospital, Chinese Academy of Medical Sciences & Peking Union Medical College, Beijing, 100730 China; 2 Department of Nephrology, Peking Union Medical College Hospital, Chinese Academy of Medical Sciences & Peking Union Medical College, Beijing, 100730 China

**Keywords:** Acute kidney injury, Acute tubular necrosis, Dehydration-associated CKD, Ischemic renal disease, Mesoamerican nephropathy

## Abstract

**Background:**

Mesoamerican nephropathy (MeN) is a pattern of chronic kidney disease (CKD) prevalent among Central American men who work in agriculture, and its underlying cause has not been elucidated. Currently, experts hypothesize that MeN is related to repeated episodes of occupational heat stress leading to water loss and hence it is also called dehydration-associated CKD.

**Case presentation:**

We report a case of a 40-year-old man, whose first admission to Peking Union Medical College Hospital was due to acute kidney injury (AKI). The clinical and pathological processes were consistent with acute tubular necrosis (ATN). However, after full recovery, CKD developed 1 year later. The second renal biopsy showed characteristics of ischemic renal disease but there was no evidence of vascular disease. It is worth noting that the patient had been taking part in long-distance running without drinking adequate water for years, which would have markedly decrease his renal blood flow. Thus, this patient may have developed chronic dehydration-associated kidney disease sharing the similar etiology of MeN.

**Conclusions:**

We report here a case of dehydration-associated CKD in a Chinese patient which shared similar etiology to MeN. Even in non-agricultural areas, this etiology of CKD should be noted to obtain a relevant history and prompt diagnosis.

## Background

In recent years, a cluster of chronic kidney disease (CKD) of unknown origin has emerged among agricultural workers, as well as in other manual laborers in various regions of the world, which is known as Mesoamerican nephropathy (MeN) [1]. This disease can not be attributed to the classic causes of kidney disease (e.g., diabetes mellitus, hypertension and glomerular diseases). Clinically, patients may present with normal or mildly elevated systemic blood pressure, reduced glomerular filtration rate, low-grade non-nephrotic proteinuria and electrolyte abnormalities [2]. Kidney biopsies have demonstrated evidence of both acute injury (acute tubular cell injury, interstitial edema, early fibrosis) and chronic injury (tubular atrophy, interstitial fibrosis) [3]. The exact etiology is unknown. The most likely cause is repeated episodes of acute kidney injury (AKI) related to dehydration and hence some researchers have named the disease dehydration-associated CKD [4]. We present a 40-year-old man who progressed from AKI to CKD sharing a similar pathogenesis to dehydration-associated CKD.

## Case presentation

A 40-year-old Chinese Han male presented to the emergency room of Peking Union Medical College Hospital with the chief complaints of nausea, vomiting and anuria. He had been in his usual health until approximately 10 days ago, when he took 500 mg paracetamol to alleviate a headache. Later in the day, nausea and vomiting developed without fever, rash, edema or gross hematuria. During the next week, his blood creatinine level increased gradually from normal to 700 μmol/L, followed by a decrease in urine output. The day before admission his urine volume was only 40 mL/day. His plasma creatine kinase levels were constantly normal.

He had a 12-year history of hypertension, with the highest blood pressure ever observed being 150/110 mmHg. His blood pressure was well controlled with amlodipine (below 140/90 mmHg). He had no significant past or family history of kidney diseases. The patient is a military man and has a comprehensive health checkup every year. A health checkup performed 1 months before the onset of his symptoms showed that his routine urine test and serum creatinine level were normal. He had been taking part in long-distance running almost every day for some years, and running approximately ten kilometers each day. Although he sweated a lot, he insisted on not drinking water during or within one hour after the exercise, aimed at “losing weight”.

Physical examination revealed an anxious appearance. He was hypertensive with a blood pressure of 148/108 mmHg (without taking his anti-hypertensive medicine on that day), respiratory rate of 20 breaths/min, and heart rate of 70 beats/min. His oxygen saturation was 97% in room air. His body mass index was 24.6 kg/m^2^. No other findings were remarkable.

His serum creatinine level was 860 μmol/L, and the urea was 12.49 mmol/L. Urinalysis showed that the white blood cell count was 15 cells/mL and red blood cell count was 80 cells/mL (only 30% were dysmorphic). Protein excretion rate was 250 mg/24 h. Ultrasound of the urinary system showed enlarged kidneys (right kidney 13.3 × 6.6 × 7.3 cm; left kidney 14.2 × 6.5 × 6.1 cm).

**Fig. 1:**
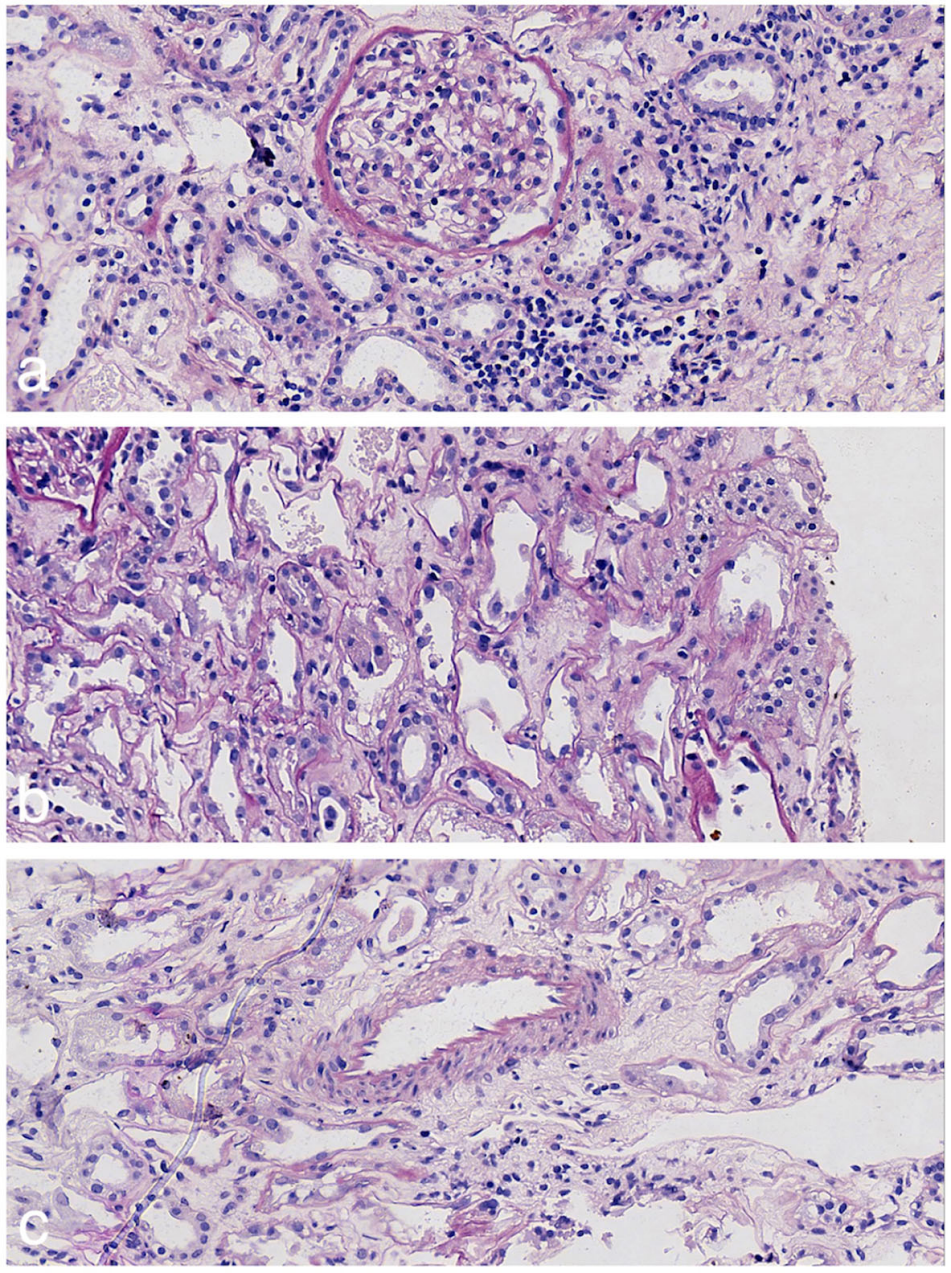
Pathological features of the first renal biopsy specimen: **a** The glomeruli shows no specific features (periodic acid-Schiff stain, 400 ×). **b** Edema of the interstitium and exposure of the basement membrane can be observed (periodic acid-Schiff stain, 400 ×). **c** The vascular structure is basically normal (periodic acid-Schiff stain, 400 ×)

The patient underwent ultrasound-guided transdermal renal biopsy. Acute tubular necrosis (ATN) was diagnosed, characterized by dilated tubules lined by flattened tubular cells, effacement of the proximal tubule brush border, distal tubule casts, and interstitial edema under the light microscopy. No tubular crystals were identified by polarized light microscopy. The glomerulus and blood vessels were largely normal (Fig. 1). Immunofluorescence and electron microscopy supported the diagnosis of ATN.

After 7 weeks of dialysis, his baseline creatinine decreased to 300 μmol/L, and serum electrolytes were normal. His urine volume increased to more than 3 L/day.

The patient’s creatinine declined continuously and stabilized at 45 μmol/L after discharge from hospital. His urinalyses were also normal. He restricted his salt and calorie intake, and started long-distance running once again, without taking enough water. He had lost 10 kg within the next year. His blood pressure kept normal without antihypertensive therapy. One year after the first discharge, a routine health checkup showed that his creatinine had increased to 150 μmol/L, and he was re-admitted to our hospital.

His routine urine test and urine sediment analysis were normal. Protein excretion rate was 420 mg/24 h. Laboratory tests including complete blood count, serum uric acid, serum immunoglobulins, complements, anti-neutrophil cytoplasmic antibodies, anti-nuclear antibodies, anti-glomerular basement membrane antibody, erythrocyte sedimentation rate and C-reactive protein were all normal. Serum tumor markers and serum immunofixation electrophoresis were also normal. Ultrasound showed that both kidneys had shrunk (right kidney 9.5 × 5.7 × 4.4 cm; left kidney 10.4 × 5.6 × 5.5 cm). Artery ultrasound including renal, carotid, lower extremity and mesenteric arteries showed no evidence of stenosis or thrombosis.

**Fig. 2:**
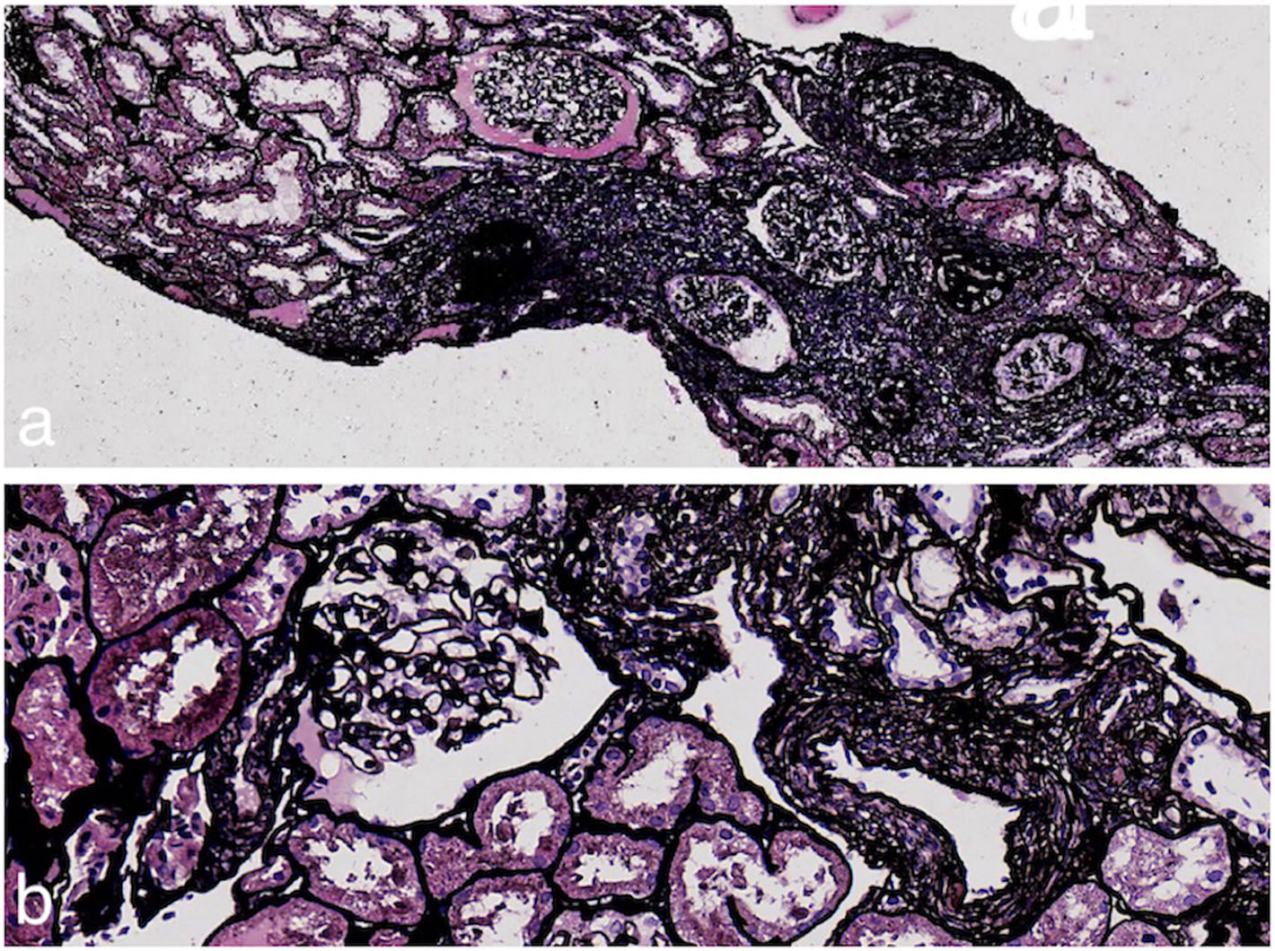
Pathological features of the second renal biopsy specimen: **a** Clearly demarcated focal injury of the glomerulus and tubules can be seen (periodic acid-Schiff-methenamine stain, 80 ×). **b** The capillary loops are basically normal (periodic acid-Schiff-methenamine stain, 400 ×)

Renal biopsy was performed for the second time. Nine of 25 glomeruli showed global sclerosis, and the basement membrane in non-sclerotic glomeruli had variable thickening and wrinkling. Patchy tubular atrophy and interstitial fibrosis were observed. The arteriolar wall was thickened and the vascular lumen was narrowed (Fig. 2), and no tubular crystals were found under polarized light. Immunoglobulins and complements were not observed in the tubulointerstitium, glomeruli, nor blood vessels under immunofluorescence. Electron microscopy showed no abnormalities.

Considering the patient’s history of long-distance running, ischemic changes in renal biopsy, and exclusion of other likely causes of CKD, especially vascular diseases, chronic dehydration-associated kidney disease similar to MeN was suspected. The patient underwent CKD management including regular monitoring, blood pressure control and lipid control. During the follow-up period, the patient’s creatinine fluctuated between 150 and 180 μmol/L.

## Discussion and conclusion

The patient was initially admitted to our hospital due to AKI. The clinical and pathological processes were consistent with ATN, which was probably associated with the use of the nephrotoxic drug paracetamol and dehydration caused by vomiting. Although paracetamol is safer for kidney than the conventional non-steroidal anti-inflammatory drugs (NSAIDs) such as ibuprofen and loxoprofen, it can lead to ATN even at therapeutic doses [5, 6]. The risk of renal toxicity potentially increases with volume depletion. After full recovery of AKI, the patient developed CKD only 1 year later, which must have involved other underlying reasons. The second renal biopsy suggested characteristics of ischemic glomeruli disease and mild vascular disease. As the patient had a 12-year history of hypertension, nephrosclerosis caused by long-term hypertension was also suspected. However, the patient had no history of malignant hypertension, and benign nephrosclerosis itself did not explain the comparatively rapid disease course. No other common etiologies were identified after thorough examinations.

A number of studies have shown that patients who have had transient AKI do have an increased risk of developing CKD/end-stage renal disease [7–9]. Considering the repeated dehydration in this patient due to heavy physical exercise without consuming sufficient fluids, chronic dehydration-associated kidney disease was considered in this case, which was similar to cases observed in rural residents in Central America .

It was reported that nephropathy mainly affected young male workers engaged in sugarcane cultivation, and was later reported in other types of agriculture. The affected individuals were asymptomatic, and had normal or only slightly elevated blood pressure and normal blood glucose levels. Urinalysis showed no or only a small amount of proteinuria (< 1 g/24 h) and small numbers of red cells and leukocytes [10]. At a workshop in Costa Rica in 2012, the disease was named MeN. In sugarcane workers, serum creatinine levels increased during their work shift. In some cases, an asymptomatic elevation in serum creatinine levels may represent a decrease in renal perfusion associated with dehydration, which, if repeated, may make the individual susceptible to CKD [11, 12].

A spate of CKD of unknown origin has also been identified in farmers in the North Central Province of Sri Lanka, central India, southern Egypt and the Sudan [1]. Similar to MeN, most patients had asymptomatic elevated serum creatinine levels, no or minimal proteinuria, and chronic interstitial nephritis with variable glomerulosclerosis [13–16]. As in MeN, patients with these diseases may initially develop AKI [17].

Renal biopsy samples from MeN patients showed chronic tubulointerstitial disease with tubular atrophy and fibrosis. Vascular changes were mild but glomerular ischaemia and global glomerulosclerosis were conspicuous. It was reported that immunofluorescence and electron microscopy analysis showed no evidence of immune complex deposits in the biopsy samples [2]. An obvious clue to the etiology of MeN is glomerular ischemia, which suggests a decrease in renal blood flow, and the absence of vascular disease may help to exclude hypertension as a primary cause [4]. One of the major clinical risk factors for the disease is working in extremely hot environment, in which dehydration often occurs. This association, coupled with the frequent use of NSAIDs by farmers, increases the likelihood of chronic recurrent kidney ischemia. Dehydration can enhance kidney damage by increasing the reabsorption of toxins in the context of volume depletion [14]. Researchers have reported that repeated dehydration caused CKD in animals [4, 18]. Other possible mechanisms leading to acute or chronic renal injury include clinical or subclinical rhabdomyolysis, elevation in serum urate level and urate crystalluria, the release of vasopressin, and activation of aldose reductase in the kidney, which generates oxidative stress [19, 20].

In our patient, we found that pathology of the first renal biopsy was consistent with ATN. The second renal biopsy showed characteristics of chronic tubulointerstitial change and ischemic renal disease. The differences between the two biopsy specimens suggested that we captured a dynamic disease process. Considering that the risk factors and pathological manifestations in our case are consistent with MeN, we believe that they share a similar pathogenesis.

Renal blood flow decreases during exercise due to the redistribution of circulation preferentially toward the muscles, heart and lungs. Several studies have demonstrated that transient AKI is very common in marathon runners and their renal function usually recovers within a short time [21, 22]. However, there is no published evidence of an increase in CKD prevalence among long-distance runners. We think that the wrong running habit played a key role in the disease pathogenesis in this patient. Appropriate water and electrolyte intake is critical in runners.

This case may represent the presentation of a larger entity—dehydration-associated CKD which may prove to be not only a newly recognized cause of CKD in Central America, but also in other regions. It is important to acquire the dehydration history in patients with unexplained ischemic nephropathy.

## Data Availability

The datasets related to this case report are available from the corresponding author.

## Abbreviations

AKI: Acute kidney injury
ATN: Acute tubular necrosis
CKD: Chronic kidney disease
MeN: Mesoamerican nephropathy
NSAIDs: Non-steroidal anti-inflammatory drugs

## References

[1] Johnson RJ, Wesseling C, Newman LS (2019). Chronic kidney disease of unknown cause in agricultural communities. N Engl J Med.

[2] Wijkstrom J, Leiva R, Elinder CG, Leiva S, Trujillo Z, Trujillo L (2013). Clinical and pathological characterization of Mesoamerican nephropathy: a new kidney disease in Central America. Am J Kidney Dis.

[3] Fischer RSB, Vangala C, Truong L, Mandayam S, Chavarria D, Granera Llanes OM (2018). Early detection of acute tubulointerstitial nephritis in the genesis of Mesoamerican nephropathy. Kidney Int.

[4] Johnson RJ, Sanchez-Lozada LG (2013). Chronic kidney disease: Mesoamerican nephropathy--new clues to the cause. Nat Rev Nephrol.

[5] Gabriel R, Caldwell J, Hartley RB (1982). Acute tubular necrosis, caused by therapeutic doses of paracetamol?. Clin Nephrol.

[6] Kato H, Fujigaki Y, Inoue R, Asakawa S, Shin S, Shima T (2014). Therapeutic dose of acetaminophen as a possible risk factor for acute kidney injury: learning from two healthy young adult cases. Intern Med.

[7] Coca SG, Singanamala S, Parikh CR (2012). Chronic kidney disease after acute kidney injury: a systematic review and meta-analysis. Kidney Int.

[8] Chawla LS, Kimmel PL (2012). Acute kidney injury and chronic kidney disease: an integrated clinical syndrome. Kidney Int.

[9] Bedford M, Farmer C, Levin A, Ali T, Stevens P (2012). Acute kidney injury and CKD: chicken or egg?. Am J Kidney Dis.

[10] Ramirez-Rubio O, McClean MD, Amador JJ, Brooks DR (2013). An epidemic of chronic kidney disease in Central America: an overview. Postgrad Med J.

[11] Wesseling C, Aragon A, Gonzalez M, Weiss I, Glaser J, Bobadilla NA (2016). Kidney function in sugarcane cutters in Nicaragua--A longitudinal study of workers at risk of Mesoamerican nephropathy. Environ Res.

[12] Sorensen CJ, Butler-Dawson J, Dally M, Krisher L, Griffin BR, Johnson RJ (2019). Risk factors and mechanisms underlying cross-shift decline in kidney function in Guatemalan sugarcane workers. J Occup Environ Med.

[13] Wijkstrom J, Jayasumana C, Dassanayake R, Priyawardane N, Godakanda N, Siribaddana S (2018). Morphological and clinical findings in Sri Lankan patients with chronic kidney disease of unknown cause (CKDu): similarities and differences with Mesoamerican nephropathy. PLoS One.

[14] Jayasumana C, Orantes C, Herrera R, Almaguer M, Lopez L, Silva LC (2017). Chronic interstitial nephritis in agricultural communities: a worldwide epidemic with social, occupational and environmental determinants. Nephrol Dial Transplant.

[15] Ganguli A (2016). Uddanam nephropathy/regional nephropathy in India: preliminary findings and a Plea for further research. Am J Kidney Dis.

[16] El Minshawy O (2011). End-stage renal disease in the El-Minia governorate, upper Egypt: an epidemiological study. Saudi J Kidney Dis Transpl.

[17] Badurdeen Z, Nanayakkara N, Ratnatunga NV, Wazil AW, Abeysekera TD, Rajakrishna PN (2016). Chronic kidney disease of uncertain etiology in Sri Lanka is a possible sequel of interstitial nephritis!. Clin Nephrol.

[18] c Markovi BL, Arambasic MD (1971). Experimental chronic interstitial nephritis compared with endemic human nephropathy. J Pathol.

[19] Butler-Dawson J, Krisher L, Asensio C, Cruz A, Tenney L, Weitzenkamp D (2018). Risk factors for declines in kidney function in sugarcane Workers in Guatemala. J Occup Environ Med.

[20] Paula Santos U, Zanetta DM, Terra-Filho M, Burdmann EA (2015). Burnt sugarcane harvesting is associated with acute renal dysfunction. Kidney Int.

[21] Mansour SG, Verma G, Pata RW, Martin TG, Perazella MA, Parikh CR (2017). Kidney injury and repair biomarkers in Marathon runners. Am J Kidney Dis.

[22] Mansour SG, Martin TG, Obeid W, Pata RW, Myrick KM, Kukova L (2019). The role of volume regulation and thermoregulation in AKI during Marathon running. Clin J Am Soc Nephrol.

